# Functional validation of the *Plasmodium falciparum* K13 C580Y mutation in recently collected Ethiopian isolates

**DOI:** 10.1128/aac.00495-26

**Published:** 2026-09-09

**Authors:** Angana Mukherjee, Ashenafi Bahita Assefa, Christopher V. Turlo, Lisa Checkley Needham, Douglas Shoue, Tarrick Qahash, Mahlet Belachew, Dagimawie Tadesse, Enirsie Kassie, Mulu Berihun, Bokretsion G. Brhane, Jonathan B. Parr, Michael T. Ferdig, the MAREE Consortium, Sisay Adane

**Affiliations:** 1 Ethiopian Public Health Institute, Addis Ababa, Ethiopia; 2 Institute for Global Health and Infectious Diseases, University of North Carolina at Chapel Hill, Chapel Hill, North Carolina, USA; 3 Department of Pathology and Laboratory Medicine, Brown University, Providence, Rhode Island, USA; 1 Department of Biological Sciences, University of Notre Dame 6111 , Notre Dame, Indiana, USA; 2 Institute for Global Health and Infectious Diseases, University of North Carolina at Chapel Hill 622440, Chapel Hill, North Carolina, USA; 3 Ethiopian Public Health Institute 128164, Addis Ababa, Ethiopia; 4 College of Medicine and Health Sciences, Arba Minch University 128157 , Arba Minch, Ethiopia; The Children's Hospital of Philadelphia, Philadelphia, Pennsylvania, USA

**Keywords:** *Plasmodium falciparum*, kelch13 (K13) C580Y, artemisinin resistance, CRISPR-Cas9, ring-stage survival assay, Ethiopia, parasite genetic background

## Abstract

Recent genomic investigation in Ethiopia identified the first detection of the *Plasmodium falciparum* Kelch13 (K13) C580Y substitution in the Horn of Africa. To assess its functional impact, we introduced C580Y into two recently collected Ethiopian clinical isolates using CRISPR-Cas9 genome editing. Ring-stage survival assays showed significantly elevated *in vitro* dihydroartemisinin survival in edited parasites relative to isogenic controls, demonstrating that C580Y confers artemisinin tolerance in contemporary Ethiopian parasite genetic backgrounds.

## INTRODUCTION

A recent genomic investigation in northwestern Ethiopia reported the first detection of the *Plasmodium falciparum* Kelch13 (K13) C580Y substitution in the Horn of Africa, a validated molecular marker of artemisinin partial resistance (ART-R). Following its emergence in western Cambodia in the late 2000s, K13 C580Y rapidly spread (1, 2) across the Greater Mekong Subregion, reaching near fixation in several countries and displacing other resistance-associated variants. Its repeated association with robust ART-R and efficient spread in low-transmission settings under dihydroartemisinin (DHA)–piperaquine selection highlights its evolutionary success (3, 4). For a K13 substitution to become prevalent in a parasite population, it must satisfy two minimal criteria: it must confer sufficient ART-R to provide a selective advantage under drug pressure, and it must remain compatible with asexual growth and transmission fitness. The evolutionary success of the K13 C580Y variant in Southeast Asia may reflect this balance.

Despite the widespread emergence of multiple K13 substitutions in Africa (5–12), C580Y has remained rare. Zeleke et al. recently identified two C580Y mutant infections from the Gondar Zuria district in northwestern Ethiopia, collected between November 2022 and October 2023 and confirmed by molecular inversion probe sequencing and long-read whole-genome sequencing. Haplotype analysis indicated a distinct genetic background relative to the Southeast Asian C580Y lineages, marking the first detection of this mutation in the Horn of Africa to our knowledge (13).

This rare detection raises an immediate biological question: does C580Y confer the expected ART-R phenotype in contemporary Ethiopian parasite genetic backgrounds? Although C580Y became predominant across much of Southeast Asia, its limited emergence in Africa may reflect differences in parasite genetic background, fitness, or selective pressures. Functional evaluation in recently collected Ethiopian parasites, rather than long-adapted laboratory lines, is therefore needed to determine whether the mutation produces the established resistance phenotype in this distinct epidemiological and genetic context. Such functional evaluation is particularly important because the ART-R phenotype associated with K13 substitutions is strongly context dependent. For example, CRISPR-Cas9 introduction of the prevalent Ugandan K13 substitutions A675V and C469Y into both Southeast Asian (Dd2) and East African (MAS-136) parasite lines showed that these substitutions did not uniformly confer reduced DHA susceptibility, particularly in the African background (14). These findings indicate that such K13 substitutions alone may be insufficient to generate resistance, and that permissive genetic backgrounds or additional genetic modifiers are required for phenotypic changes. The introduction of K13 C580Y produced variable phenotypes in different genetic backgrounds, conferring resistance in Cambodian, Dd2 (15), Vietnamese (V1/S) (16), Ugandan, and 3D7 (17) isolates but failed to do so in others, such as Tanzanian (F32) (17) and Chinese (FCC1/HN) (18)isolates. Collectively, these studies demonstrated that the ART-R phenotypic consequences of K13 substitutions cannot be inferred from their presence alone; they must be evaluated in the context of relevant genetic backgrounds from the same region or country.

**Fig 1:**
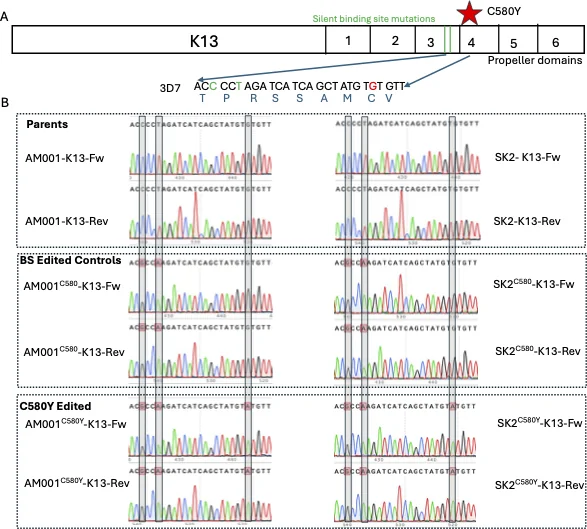
CRISPR-Cas9 genetic editing of K13 C580Y and silent binding site control mutations in AM001 and SK2 parasites. (**A**) Locations of the C580Y and control substitutions in the K13-propeller domains. The reference DNA sequence of 3D7 is shown below. (**B**) Sanger sequence and chromatogram analysis of forward and reverse alleles of parent and edited lines. The highlighted bases show the WT and edited alleles.

We investigated the impact of K13 C580Y by introducing it into two recently collected clinical isolates of Ethiopian *P. falciparum* and assessed ART-R *in vitro*. The isolates, AM001 (Arba Minch, South Ethiopia Regional State) and SK2 (Selekleka, Tigray, northern Ethiopia), were collected in 2024. Venous blood (5–7 mL) from *P. falciparum*-infected patients was collected in sterile heparinized tubes, washed with incomplete RPMI medium, and cryopreserved in glycerolyte within 24 h of collection as per the WWARN guidelines. The isolates were subsequently culture-adapted *in vitro*. After thawing, AM001 and SK2 adapted readily to *in vitro* culture conditions and were expanded for approximately four (AM001) and three (SK2) asexual replication cycles before initial cryopreservation as laboratory stocks. Frozen stocks were then thawed to confirm recovery after a laboratory freeze–thaw cycle and expanded for approximately four to five additional cycles before use in downstream *in vitro* assays and transfection experiments. Both were confirmed to carry wild-type K13 propeller sequences using Sanger sequencing (Fig. 1). Using CRISPR-Cas9 genome editing, we edited *k13* for C580Y in both the isolates. As editing controls, we also introduced two synonymous shield/binding site mutations in *k13* that did not alter the amino acid sequences (C580) (19). Editing of *k13* was performed using the pDC2-coSpCas9-k13guide-gRNA-h*dhfr* all-in-one plasmid containing a *P. falciparum* codon-optimized Cas9 sequence, a human dihydrofolate reductase (h*dhfr*) gene expression cassette (conferring resistance to WR99210), and a donor template (containing the respective non-synonymous and shield/binding site mutations or only shield/binding site mutations) (20). Eighty microliters of packed RBCs containing 5% ring parasitemia was electroporated with 50 µg of the purified plasmid resuspended in Cytomix using a BioRad Gene Pulser Xcell Electroporation System. Transfected parasites were maintained under 2.5 nM WR99210 (Jacobus Pharmaceuticals) to select edited parasites until 6 days post-transfection, after which the drug was removed from the media. Recrudescent parasites were first detected by microscopy at 28 days post-electroporation for the AM001^C580Y^ and AM001^C580^ cultures and at 27 days post-electroporation for the SK2^C580Y^ and SK2^C580^ lines; cultures were subsequently expanded for molecular confirmation, freezing, and downstream phenotyping.

**Fig 2:**
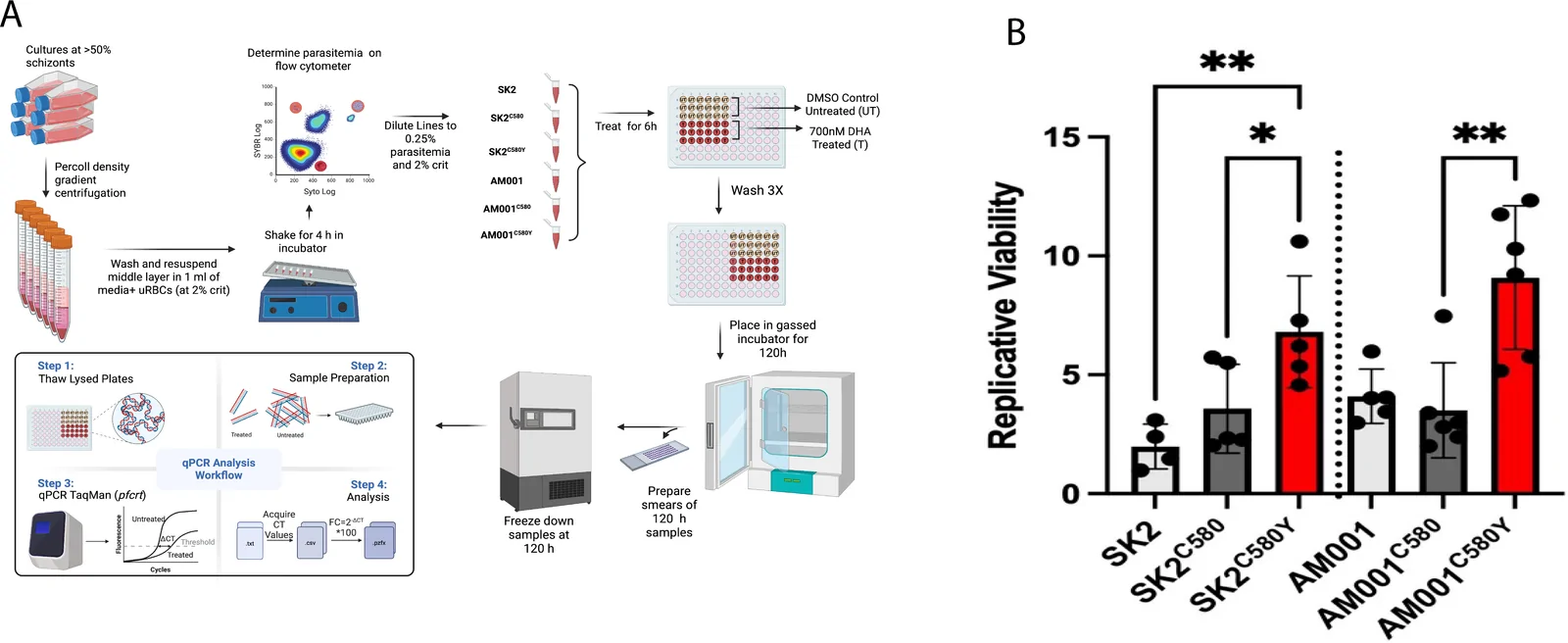
Experimental workflow and DHA survival phenotype of K13-edited Ethiopian isolates. (**A**) Schematic of the ring survival assay. Mature schizonts were synchronized by 75% Percoll and allowed to invade fresh red blood cells for 4 h to form early ring-stage parasites. Early ring-infected RBCs were adjusted to 0.2% parasitemia and 2% hematocrit. For each parasite line, parallel matched cultures were exposed to either 700 nM DHA or dimethyl sulfoxide (DMSO, vehicle control) for 6 h in 96-well plates. Cultures were then washed to remove the drug and maintained in drug-free medium for 120 h, after which parasite abundance was quantified by SYBR Green-based qPCR. Replicative viability after DHA exposure was calculated relative to the matched DMSO-treated culture for the same parasite line. (**B**). Replicative viability of parental, silent-edited control, and C580Y-edited parasites in the AM001 and SK2 genetic backgrounds following DHA exposure. For each parasite line, each biological replicate consisted of matched parallel cultures treated with either 700 nM DHA or DMSO vehicle control. Replicative viability was calculated as parasite abundance after DHA exposure relative to the matched DMSO-treated control for the same parasite line. Four to six independent biological replicates were performed for each parasite line. Bars represent mean replicative viability, and error bars indicate SEM. Statistical comparisons were performed on DHA-normalized replicative viability values within each genetic background using unpaired *t*-tests with Welch’s correction, comparing the C580Y-edited line separately with its matched silent-edited control and corresponding parental line: AM001^C580Y^ vs AM001^C580^, AM001^C580Y^ vs AM001, SK2^C580Y^ vs SK2^C580^, and SK2^C580Y^ vs SK2. The AM001^C580Y^ vs AM001 parental comparison was not statistically significant. Significant comparisons are indicated: *P < 0.05, **P < 0.005.

Successful editing was confirmed using Sanger sequencing (Fig. 1). To our knowledge, this represents one of the first functional validations of a K13 substitution performed through genome editing in recently collected African patient isolates. DHA susceptibility was then assessed using a DHA survival screen performed at 700 nM DHA with a qPCR readout at 120 h post-exposure (Fig. 2A) (21). C580Y-edited parasites showed significantly elevated survival than their respective isogenic controls in both backgrounds (SK2: *P* = 0.0445, AM001: *P* = 0.0031; unpaired Welch’s *t*-test) (Fig. 2B). These findings demonstrate that C580Y is sufficient to confer increased artemisinin tolerance in two Ethiopian parasite backgrounds examined and provide functional context for interpreting its recent detection by Zeleke et al. The increased DHA survival observed in both AM001 and SK2 indicates that the effect of C580Y is reproducible across two distinct Ethiopian parasite backgrounds. Designed as a gain-of-function analysis, the study did not seek to identify the specific background variants that may modulate the magnitude of the phenotype. Reediting the engineered C580Y lines back to the wild-type C580 allele would provide an additional test of causality, but this experiment was not performed.

Together, these findings raise concern that C580Y in Ethiopia could contribute to clinically relevant ART-R, as previously observed in Southeast Asia. Determining the asexual growth and transmissible fitness of these isogenic C580Y-edited lines will be essential to assess whether this newly emergent mutation is likely to persist locally or expand. Such studies will be critical for distinguishing transient emergence from evolutionary success and for anticipating the public-health significance of C580Y in Africa.
